# Genome-Wide Identification and Expression Analysis Under Abiotic Stress of the *Lipoxygenase* Gene Family in Maize (*Zea mays*)

**DOI:** 10.3390/genes16010099

**Published:** 2025-01-18

**Authors:** Sinan Li, Shuai Hou, Yuanqing Sun, Minghao Sun, Yan Sun, Xin Li, Yunlong Li, Luyao Wang, Quan Cai, Baitao Guo, Jianguo Zhang

**Affiliations:** Heilongjiang Academy of Agricultural Sciences, Harbin 150086, China; lee18686774002@126.com (S.L.);

**Keywords:** maize, LOX, abiotic stress, gene family, expression analysis

## Abstract

**Background/Objectives:** Abiotic stresses impose significant constraints on crop growth, development, and yield. However, the comprehensive characterization of the maize (*Zea mays*) *lipoxygenase* (*LOX*) gene family under stress conditions remains limited. LOXs play vital roles in plant stress responses by mediating lipid oxidation and signaling pathways. **Methods:** In this study, 13 *ZmLOX* genes were identified in maize and characterized to explore their functions under abiotic stresses. **Results:** Phylogenetics revealed that *ZmLOX* genes share evolutionary origins with *LOX* genes in Arabidopsis and rice. Promoter analysis identified *cis*-acting elements associated with growth, light response, hormone signaling, and stress response, indicating their diverse biological roles. Gene Ontology (GO) and KEGG enrichment analyses showed that *ZmLOX* genes are involved in jasmonic acid metabolism, lipid signaling, and photosynthetic processes, while protein–protein interaction (PPI) analysis positioned ZmLOX proteins as central hubs in stress-related regulatory networks. Differential expression and qRT-PCR analyses revealed stress-specific (including heat, drought, salt, and cold) expression patterns, with *ZmLOX2* and *ZmLOX13* showing key roles in drought and cold tolerance, respectively. **Conclusions:** These findings provide new insights into the regulatory functions of *ZmLOX* genes, offering potential targets for enhancing maize resilience to abiotic stresses and improving agricultural productivity.

## 1. Introduction

A lipoxygenase (LOX) is a kind of peroxide dioxygenase that contains non-heme ferritin, does not contain sulfur, and is composed of a single polypeptide chain. It is widely found in plants and animals. LOXs are widely found in plants and initiate the synthesis of a series of cyclic or aliphatic compounds, which is often referred to as the LOX pathway or the octadecanoic pathway [1]. LOX pathway metabolites in plants have a wide range of physiological functions. These metabolites play an active role in plant growth and development and defense response to stress, such as inducing cell senescence and death, mechanical damage, and biotic and abiotic stresses [2,3].

LOX proteins contain a non-heme iron as the catalytic component of the active center, and its active state is the oxidized ferric ion (Fe^3+^). One water molecule and five (the five ligands are oxygen on the carboxyl group of isoleucine or valine at the carbon terminus, nitrogen on two histidine residues of *α*-helix 9, nitrogen on one histidine residue of *α*-helix 18, and an oxygen atom on the distal end of asparagine or serine residues of *α*-helix 18) amino acid residues are used as ligands to form a six-coordinated octahedral structure with the non-heme iron [4]. LOX proteins generally contain about 38 conserved amino acid residues, of which histidine is essential for LOX to bind iron ions and plays an important role in the catalytic activity of LOX [5].

The *LOX* can be divided into *9-LOX* and *13-LOX* according to the oxygenation sites catalyzed by *LOX*, but some *LOXs* can be oxygenated at both sites to produce two products; this *LOX* is called *9/13-LOX*, such as *OsLOX1* in rice (*Oryza sativa*) and *LOXN2* in pea (*Pisum sativum* L.) [6,7]. Previous studies have shown that *LOX* plays an important role in plant growth and development and stress [8,9]. In plants, *9-LOX* mainly plays a role in organ development and fruit-ripening processes [1], and *13-LOX* is mainly involved in the defense response to biotic and abiotic stresses [10,11]. For example, 17 *LOX* genes were identified in potato (*Solanum tuberosum*), of which two *StLOX* genes (encoding 13-LOX protein) were involved in salt stress response [12]. In addition, 72 *LOX* genes were identified in cultivated peanuts (*Arachis hypogaea*) and wild peanuts (*Arachis duranensis* and *Arachis ipaensis*), and overexpression of *AhLOX29* in Arabidopsis (*Arabidopsis thaliana*) enhanced drought stress resistance [13]. A total of 64 *LOX* genes were identified in cotton, and cotton with silencing of *GhLOX12* and *GhLOX13* showed increased sensitivity to salt stress [14]. *LOX* family genes have been identified and analyzed in a variety of plants, including potato, peanut, melon (*Cucumis melo* L.), pear (*Pyrus bretschneideri*), kiwi (*Actinidia chinensis*), cucumber (*Cucumis sativus* L.), passionflower (*Passiflora edulis*), diploid woodland strawberry (*Fragaria vesca*), artemisia annua (*Artemisia annua* L.), foxtail millet (*Setaria italica*), jujube (*Ziziphus jujuba*), sorghum (*Sorghum bicolor* L.), cotton, pepper (*Capsicum annuum* L.), tea plant (*Camellia sinensis*), peach tree (*Prunus persica*), poplar (*Populus*), Arabidopsis, and rice [12,13,14,15,16,17,18,19,20,21,22,23,24,25,26,27,28,29]. Most of these *LOX* family genes play roles in fruit aroma formation and stress response.

As one of the most extensively cultivated crops globally, maize holds immense theoretical and practical importance for mining stress-resistant genes. Previous studies have identified *LOX* genes in maize [30]; however, a comprehensive investigation of the maize *LOX* gene family is lacking. To address this knowledge gap, we conducted a genome-wide identification and analysis of maize *LOX* genes, encompassing fundamental gene information, chromosomal positioning, gene structure, encoded protein motifs, phylogenetic relationships, and collinearity. Additionally, we examined the transcriptomic data of all *ZmLOX* genes under abiotic stress conditions and verified expression patterns using qRT-PCR. Our study sheds light on the evolutionary history and functional diversification within the maize *LOX* gene family, establishing a theoretical foundation for further elucidating the regulatory mechanisms underpinning plant stress tolerance. By systematically characterizing this critical gene family, our findings contribute to the broader understanding of stress resistance in maize and pave the way for future research and applications in crop improvement.

## 2. Results

### 2.1. Identification and Analysis of LOX Family Genes in Maize

Arabidopsis LOX protein sequences were compared against the maize protein database using Blastp, and conserved domains of the LOX proteins were identified based on the HMM file. By integrating the results of protein sequence searches and Pfam domain identification, a total of 13 *LOX* genes were identified in maize. These genes were named from *ZmLOX1* to *ZmLOX13*, following their chromosomal positions. The encoded ZmLOX proteins had amino acid lengths ranging from 850 (ZmLOX7) to 968 (ZmLOX1). Their molecular weights varied between 95.9922 kDa (ZmLOX7) and 108.2720 kDa (ZmLOX1), while the predicted isoelectric points (pI) ranged from 6.0036 (ZmLOX5) to 8.5904 (ZmLOX1). Subcellular localization predictions indicated that ten ZmLOX proteins are located in the cytoplasm, while three are localized to the chloroplast (Table 1).

### 2.2. Chromosomal Distribution of ZmLOX Genes

**Figure 1:**
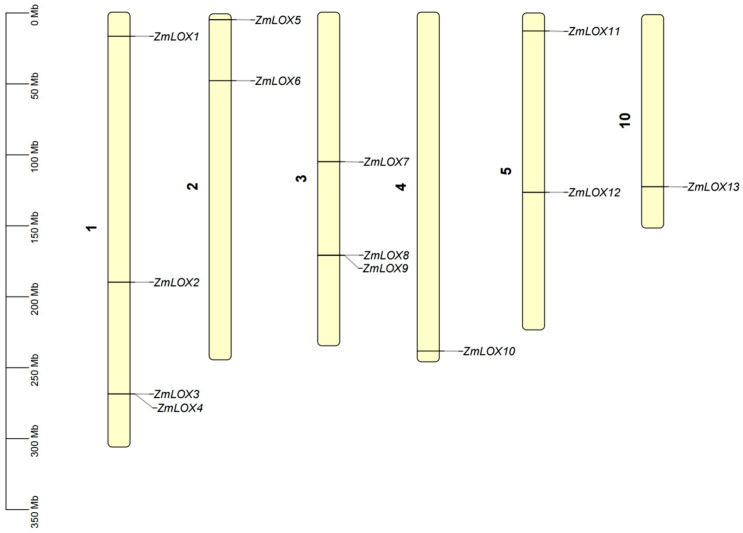
Chromosome locations of *ZmLOX* genes. The scale on the left represents the lengths of the chromosomes.The number 1 represents chromosome 1, the number 2 represents chromosome 2, and so on.

Analysis of chromosomal positions revealed that 13 *ZmLOX* genes were distributed on chromosomes 1, 2, 3, 4, 5, and 10. Chromosome 1 contains the most *ZmLOX* genes (four genes), chromosome 3 contains three *ZmLOX* genes, chromosomes 2 and 5 each contain two *ZmLOX* genes, and chromosomes 4 and 10 each contain the least *ZmLOX* genes (one gene). Moreover, our analysis revealed that the majority of *ZmLOX* genes were located in the telomeric regions of the maize chromosomes (Figure 1).

### 2.3. Characterization of Conserved Motifs and Structural Features in ZmLOX Family

**Figure 2:**
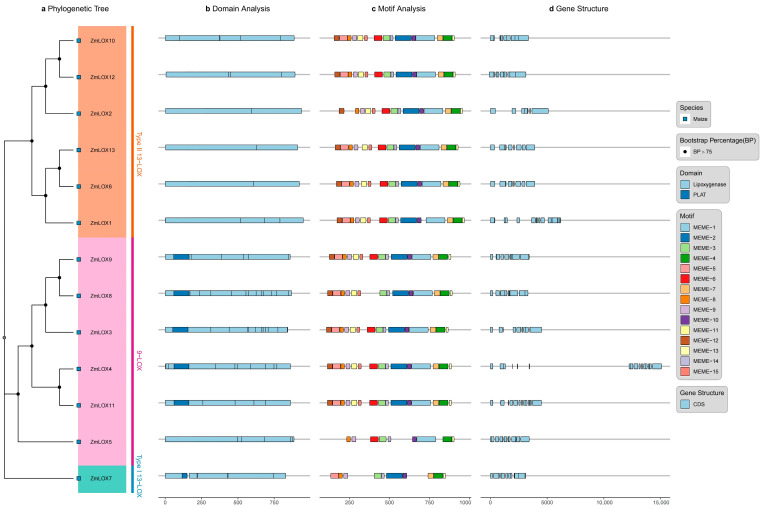
Characteristic analysis of *ZmLOX* gene family members. (**a**) Phylogenetic tree of ZmLOX proteins. (**b**) Conserved domains of ZmLOX proteins. (**c**) Motifs analysis of ZmLOX protein. (**d**) Gene structure analysis of *ZmLOX* genes.

The thirteen maize LOX proteins were categorized into two groups: 9-LOX, comprising six LOX proteins, and 13-LOX, consisting of seven LOX proteins. The 13-LOX group was further subdivided into Type I and Type II 13-LOX (Figure 2a). All identified LOX proteins were classified based on their conserved domains, including the lipoxygenase and PLAT domains (Figure 2b). A total of 15 motifs were identified across the maize ZmLOX proteins. Motifs 3, 4, 8, 9, 10, 13, 14, and 15 were shared by all ZmLOX proteins. ZmLOX5 contained nine motifs, ZmLOX7 contained ten motifs, and ZmLOX1 and ZmLOX2 contained fourteen motifs, while the remaining ZmLOX proteins contained all fifteen motifs (Figure 2c). Structural analysis of the *ZmLOX* family genes, conducted using genome annotation data, revealed that all *ZmLOX* genes contained introns (Figure 2d).

### 2.4. Phylogenetic Analysis of LOX Family Proteins

**Figure 3:**
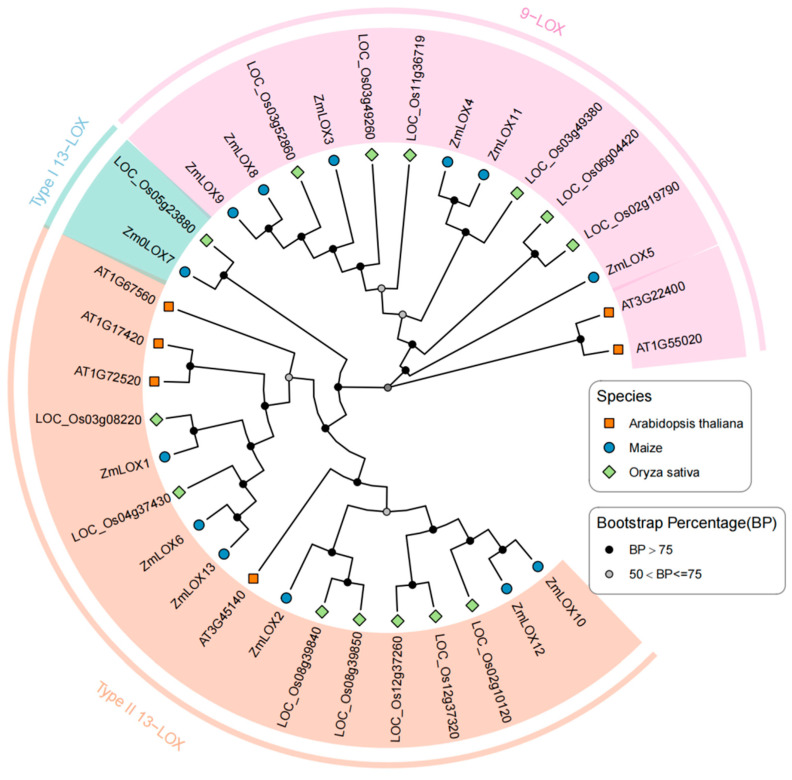
Phylogenetic tree of LOX proteins in 3 species of plants. Three groups of LOX in different plant species were marked with different shapes: rectangle means *A. thaliana*, circle means maize, and rhombus means *O. sativa*.

A phylogenetic tree was constructed based on the protein sequences of LOX family members from maize, Arabidopsis, and rice. The analysis grouped all LOX proteins into three distinct clades: 9-LOX, Type I 13-LOX, and Type II 13-LOX. The 9-LOX group consisted of six maize LOX proteins, two Arabidopsis LOX proteins, and six rice LOX proteins. The Type II 13-LOX group included six maize LOX proteins, four Arabidopsis LOX proteins, and seven rice LOX proteins. The Type I 13-LOX group was the smallest, containing one maize LOX protein and one rice LOX protein (Figure 3). These results highlight the evolutionary relationships and structural divergence of LOX proteins among the three species.

### 2.5. Collinearity Analysis of LOX Family Genes in Maize

**Figure 4:**
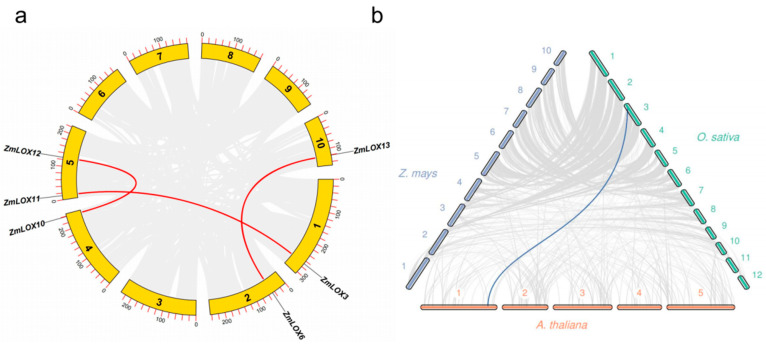
Gene duplication analysis of *ZmLOX* genes. (**a**) Collinearity analysis of *LOX* genes in maize. (**b**) Synteny analysis of *LOX* genes in maize, Arabidopsis, and rice.

Collinearity analysis was performed on all maize *LOX* genes to understand the duplication events of *ZmLOX*. The results show that *ZmLOX3* and *ZmLOX11*, *ZmLOX6* and *ZmLOX13*, and *ZmLOX10* and *ZmLOX12* have collinearity relationships (Figure 4a). To elucidate the evolutionary mechanism of *LOX* family genes in maize, we performed a synteny analysis of *LOX* genes in maize compared with those in Arabidopsis and rice. None of the *ZmLOX* genes have a synteny relationship with the Arabidopsis *LOX* genes and rice *LOX* genes. The results show that one *LOX* gene has a synteny relationship between Arabidopsis and rice (Figure 4b).

### 2.6. Cis-Acting Element Analysis of ZmLOX’s Promoters

**Figure 5:**
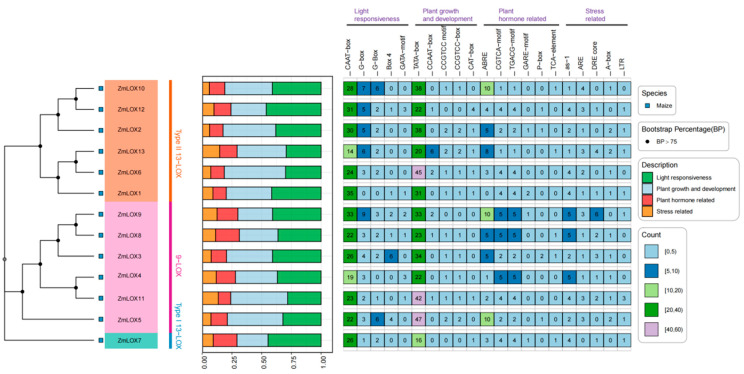
*Cis*-element analysis of ZmLOX promoters.

To identify and characterize the cis-regulatory elements, we analyzed the 1500 bp upstream sequences of the coding regions for all 13 ZmLOX genes. The discovered cis-acting elements were visualized using TBtools software (Figure 5). Our analysis revealed the presence of twenty-one distinct cis-regulatory motifs within the promoter regions of the ZmLOX gene family, categorized into five light-responsive elements, five plant growth and development elements, six phytohormone-related elements, and five stress-related elements. Notably, all *ZmLOX* promoters were enriched with plant developmental elements (e.g., TATA box) and light-responsive elements (e.g., CAAT box). Among them, the promoters of *ZmLOX7* and *ZmLOX8* exhibited a higher abundance of light-responsive elements, while the promoters of *ZmLOX4*, *ZmLOX8*, *ZmLOX11*, and *ZmLOX13* contained more stress-responsive elements, suggesting diverse regulatory roles under varying environmental conditions.

### 2.7. GO and KEGG Annotation of ZmLOX Genes

**Figure 6:**
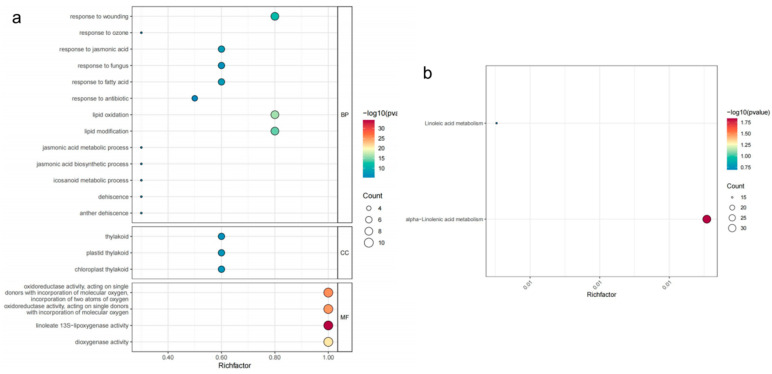
GO and KEGG enrichment of *ZmLOX* genes. (**a**) GO enrichment. (**b**) KEGG enrichment. The x-axis represents the Rich factor, indicating the proportion of genes associated with each term relative to the background set, while the size of the data points reflects the gene count and the color gradient denotes statistical significance (−log10(*p*-value)).

As shown in Figure 6, the results of Gene Ontology (GO) enrichment analysis (Figure 6a), encompass three categories: biological processes (BPs), cellular components (CCs), and molecular functions (MFs). Key enriched terms include processes such as “jasmonic acid biosynthetic process,” “response to wounding,” and “anther dehiscence” under BPs, components like “chloroplast thylakoid” and “plastid thylakoid” under CCs, and activities such as “dioxygenase activity” and “oxidoreductase activity” under MFs. The results highlight significant enrichment of terms related to jasmonic acid metabolism, lipid oxidation, and thylakoid-related components, suggesting these pathways play a critical role in the function of *ZmLOX* genes. The KEGG pathway enrichment analysis (Figure 6b) highlights two significantly enriched pathways: α-linolenic acid metabolism and linoleic acid metabolism, critical in lipid metabolism.

### 2.8. Protein–Protein Interaction Network of ZmLOX in Maize

**Figure 7:**
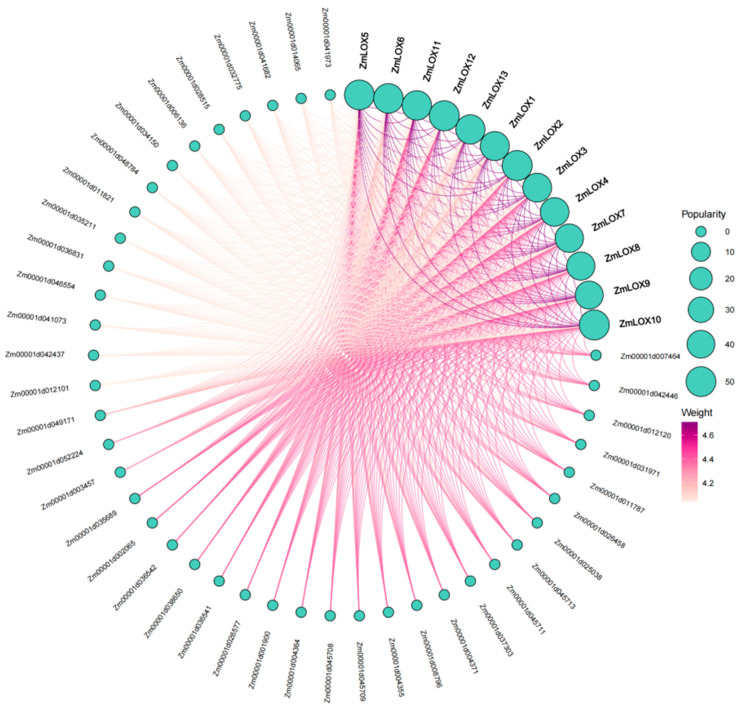
PPI network between ZmLOX proteins and other proteins in maize. Each node represents a protein and edges indicate their interactions.

To explore potential regulatory mechanisms of ZmLOX in maize, a protein–protein interaction (PPI) network was constructed. As shown in Figure 7, This PPI network analysis illustrates the interactions between key proteins. Prominent nodes, such as members of the ZmLOX family (e.g., ZmLOX5 and ZmLOX7), act as central hubs with high connectivity, suggesting their pivotal roles in regulatory or signaling pathways. In addition to ZmLOX proteins, a total of 40 proteins were identified as interacting partners of ZmLOX, and these proteins are likely to play important roles in the pathways where ZmLOX exerts its functions.

### 2.9. Differential Expression and Expression Profiling of ZmLOX Genes Based on Transcriptome Data

**Figure 8:**
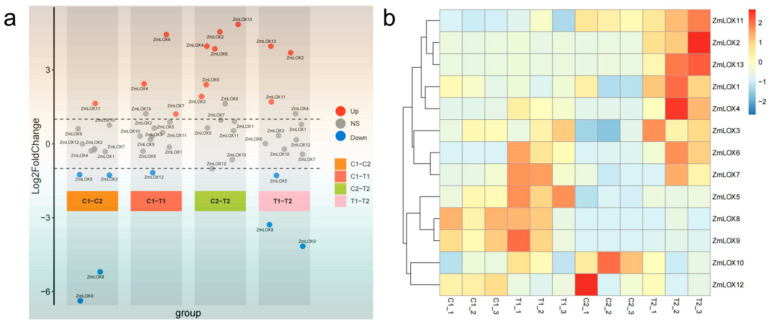
Expression profiling of *ZmLOX* genes in cold-sensitive maize and cold-resistant maize under cold treatment. (**a**) Differential expression of *ZmLOX* genes; samples mean control samples and treatment groups in differential expression gene analysis. (**b**) Expression heatmap of *ZmLOX* genes. C means control groups (25 °C), T means treatment groups (4 °C), and 1 or 2 means cold-sensitive maize and cold-resistant maize.

To analyze the differential expression and expression profiling of *ZmLOX* genes, volcano and heatmap plots were constructed. Figure 8a presents multiple volcano plots comparing the differential expression of *ZmLOX* genes across four group comparisons (C1-C2, C1-T1, C2-T2, T1-T2, same treatment or same sample type). *ZmLOX* genes are categorized as upregulated (Up, |log2FoldChange| > 1, and adj. *p* < 0.05), downregulated (Down, |log2FoldChange| > 1, and adj. *p* < 0.05), or non-significant (NS, |log2FoldChange| < 1, or adj. *p* < 0.05), with key genes such as *ZmLOX2* and *ZmLOX13* displaying significant expression changes across multiple comparisons. *ZmLOX8* and *ZmLOX9* display significant downregulated in C1-C2 and T1-T2, indicating that these exhibited expression specificity in different maize varieties. *ZmLOX4* was upregulated in the treatment group, indicating that it is the cold-responsive gene in different maize varieties. In addition, referring to Figure 8b, the results indicate that genes *ZmLOX2* and *ZmLOX13* are likely to be specifically differentially expressed in cold-tolerant variety 2 under cold treatment.

### 2.10. Expression Analysis of ZmLOX Family Genes Under Abiotic Stress Using qRT-PCR

**Figure 9:**
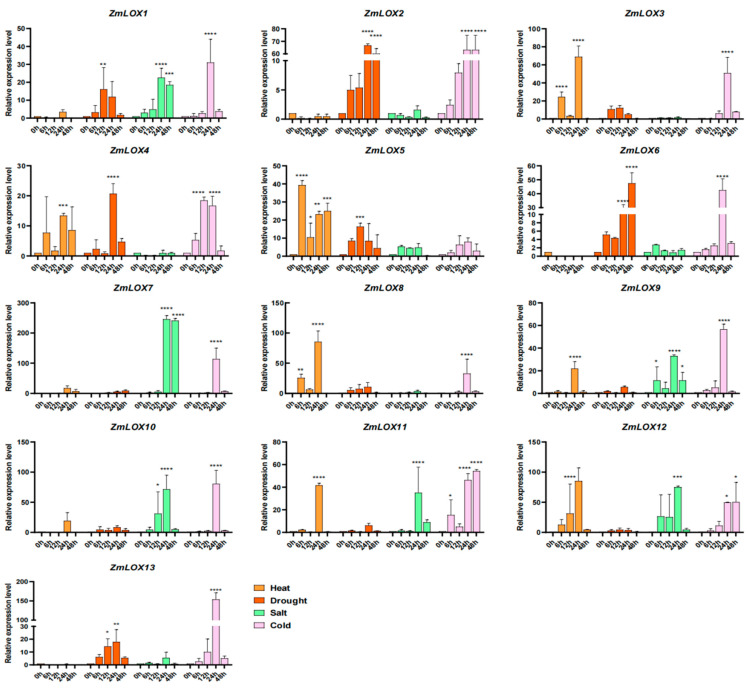
The qRT-PCR analysis of 13 *ZmLOX* genes under four types of abiotic stresses. Error bars represent the standard error of the mean (SEM; n = 3). * indicates significant difference (*p* < 0.05), ** indicates highly significant difference (*p* < 0.01), *** indicates highly significant difference (*p* < 0.001) and **** indicates extremely significant difference (*p* < 0.0001).

To analyze the responsiveness of *ZmLOX* genes to different abiotic stresses, including heat, drought, salt, and cold stress, the relative expression levels of *ZmLOX* genes were analyzed using qRT-PCR. As shown in Figure 9, seven *ZmLOX* genes exhibit significantly upregulated expression patterns in response to heat stress; except for *ZmLOX5*, the other six genes reach their highest relative expression levels at 24 h under heat stress. Six genes show significant responsive expression under drought stress, particularly *ZmLOX2*, which is upregulated nearly 70-fold after 24 h of drought treatment. Similarly, six genes display significant responsive expression patterns under salt stress; except for *ZmLOX9*, the other five *ZmLOX* genes are significantly upregulated after 12 h. Regarding cold stress, twelve ZmLOX genes show significant responsive expression patterns; except for *ZmLOX2* and *ZmLOX11*, the other ten genes exhibit an expression pattern that first increases and then decreases under cold stress.

## 3. Discussion

Maize is a kind of food crop that is widely grown in the world. Environmental conditions such as temperature, humidity, light, moisture, and biodiversity vary greatly in different planting areas. Therefore, maize is often faced with stresses such as cold, heat, drought, salt, diseases, and pests in actual agricultural production [31]. When plants are stressed, the intracellular reactive oxygen species (ROS), such as H_2_O_2_ and O_2_^−^·, will rise sharply. These excessive ROS can cause excessive oxidation of cells and damage the intracellular protein structure, which seriously affects the biological function of cells [32]. Lipoxygenase (LOX) enzymes facilitate the oxidation of polyunsaturated fatty acids, producing oxylipins via enzymatic or non-enzymatic pathways that play crucial roles in fatty acid metabolism [33]. In plants, *LOX* genes are often upregulated under stress conditions and play a regulatory role in enhancing tolerance to abiotic stresses. For instance, *PgLOX3* in ginseng (*Panax ginseng*) has been shown to positively regulate drought tolerance [34]. In rapeseed (*Brassica campestris*), various *LOX* genes respond to drought and heat stress [35]. In soybean (*Glycine max*), *LOX*-silenced plants exhibited reduced singlet oxygen (^1^O_2_) formation under osmotic stress and promoted root growth compared to wild-type plants [36]. Furthermore, when *AfLOX4*, a gene from *Amorpha fruticosa* L., was overexpressed in tobacco (*Nicotiana tabacum*), it considerably improved the plant’s resilience to NaHCO3 and drought stress conditions. [37].

Studies have demonstrated that LOX activity increases progressively with drought stress duration in maize (*Zea mays* L.). However, a comprehensive analysis of the *LOX* gene family under these conditions remains to be elucidated [2]. In plants, genes usually exist in the form of gene families. By identifying the LOX gene family in maize and investigating its structural features and expression profiles under various stress conditions, we gain an important understanding of the possible functions these genes may play. Preliminary predictions of their functions under stress are crucial for understanding their contribution to stress tolerance, offering a foundation for improving maize resistance to environmental challenges. In this study, a total of thirteen *ZmLOX* family genes were identified in maize, of which six encode 9-LOX, one encodes Type I 13-LOX, and six encode Type II 13-LOX (Table 1). The 13 *ZmLOX* genes were randomly distributed on different chromosomes, and the distribution on the same chromosome was also uneven, with most *ZmLOX* genes distributed at both ends of the chromosome (Figure 1). This phenomenon, observed in most gene family studies [12,13,14,19,20,21,23,24,25,27,29], suggests that many *ZmLOX* genes might be highly expressed and functionally active in maize. In this study, we analyzed the structure of *ZmLOX* genes and the conserved motifs of their encoded proteins (Figure 2), constructed a phylogenetic tree of ZmLOX proteins (Figure 3), and examined collinearity among *ZmLOX* genes in maize (Figure 4). The phylogenetic analysis revealed that ZmLOX proteins are grouped with LOX proteins from Arabidopsis and rice, indicating conserved evolutionary relationships. Additionally, synteny analysis showed that *LOX* genes in maize, Arabidopsis, and rice share syntenic relationships, suggesting that these genes may have originated from a common ancestor and persisted through evolutionary processes. Examination of the *cis*-regulatory elements within the promoter regions of ZmLOX genes uncovered a high prevalence of motifs related to plant growth, development, and responsiveness to light stimuli (Figure 4). This suggests that *LOX* genes might play a crucial role in plant growth and development, possibly acting as essential regulatory factors in these processes. In addition, the presence of hormone response type and stress response type elements in the promoter of the *ZmLOX* gene suggests that the *ZmLOX* family genes may have hormone or stress response functions. This pattern is similar to the *cis*-acting elements of LOX genes identified in cotton [14]. The GO and KEGG pathway enrichment analyses (Figure 6) provided key insights into the functional roles of *ZmLOX* genes. The significant enrichment of GO terms related to jasmonic acid metabolism, lipid oxidation, and thylakoid-associated components underscores the involvement of *ZmLOX* genes in critical biological processes, including stress responses and photosynthesis. Similarly, the identification of α-linolenic acid and linoleic acid metabolism pathways through KEGG enrichment highlights the importance of lipid signaling in ZmLOX-mediated functions. Complementing these findings, the PPI network (Figure 7) identified ZmLOX proteins as central hubs with extensive connectivity, interacting with 40 other proteins. These results suggest that ZmLOX proteins are pivotal in regulatory and signaling networks, mediating stress responses and other vital functions.

When exposed to stressful environments, plants frequently display alterations in gene expression patterns as an adaptive mechanism to cope with adverse conditions, potentially leading to improved stress resilience [38]. Analysis of transcriptomic datasets from stress-exposed maize populations enabled the examination of *ZmLOX* gene expression profiles under diverse environmental challenges (Figure 8). Additionally, the relative expression levels of all *ZmLOX* genes were validated under these stress conditions using qRT-PCR analysis (Figure 9). The expression profiling analyses in Figure 8 and Figure 9 further delineate the regulatory roles of *ZmLOX* genes under various conditions. Differential expression analysis (Figure 8) revealed that *ZmLOX* genes, such as *ZmLOX2* and *ZmLOX13*, exhibit specific upregulation in cold-tolerant maize varieties under cold treatment, suggesting their critical roles in cold tolerance mechanisms. Meanwhile, qRT-PCR data (Figure 9) demonstrated that *ZmLOX* genes respond distinctly to abiotic stresses, with several genes showing significant upregulation in response to heat, drought, salt, and cold stresses. Consistent with previous studies, differential accumulation of LOX has been observed among maize genotypes with varying degrees of salt/alkali sensitivity [3]. Additionally, in peanut (*Arachis hypogaea* L.), *LOX* genes have been demonstrated to be inducible by various biotic stresses [13]. Notably, *ZmLOX2* exhibited a nearly 70-fold upregulation under drought stress, and ten genes displayed transient expression peaks under cold stress, possibly reflecting a delayed participation in cold-responsive pathways. These results collectively underscore the diverse and stress-specific regulatory functions of *ZmLOX* genes, providing a foundation for further exploration of their roles in maize stress tolerance.

## 4. Materials and Methods

### 4.1. Plant Materials and Treatments

Maize material of the genotype T641 was utilized in this research. These maize materials were cultivated in a light incubator (MGC-100, Shanghai Yiheng Scientific Instrument Co., Ltd.) under a photoperiod of 16 h of light and 8 h of darkness at 25 °C until the three-leaf stage. For heat stress, the plant growth temperature was set to 35 °C. For drought stress, the cleaned maize roots were soaked with a 10% PEG6000 solution. For salt stress, the cleaned maize roots were soaked with 100 mmol/L NaCl solution. For cold stress, the plant growth temperature was set to 5 °C. The sampling time points were 0 h, 6 h, 12 h, 24 h, and 48 h, with three biological replicates established for each group.

### 4.2. Identification of LOX Family Genes in Maize

To identify LOX family members in maize, we employed a multi-step approach utilizing various bioinformatics tools and databases. First, we acquired the LOX hidden Markov model (HMM) file (PF00305) from the InterPro database (https://www.ebi.ac.uk/interpro/, accessed on 10 November 2024). We then obtained the maize genome data, including genome annotations and protein sequences, from the Phytozome database (https://phytozome-next.jgi.doe.gov/, accessed on 15 November 2024). Using the hmmsearch software (v3.3.1), we scanned the maize protein sequences against the LOX HMM file to detect potential ZmLOX genes. In parallel, we retrieved LOX protein sequences from Arabidopsis through the TAIR database (https://www.arabidopsis.org/, accessed on 15 November 2024) and performed a comparative analysis against the maize protein dataset using Blastp (v2.13.0). By integrating results from these complementary approaches, we compiled a comprehensive list of putative *LOX* family members in maize. To validate the presence of conserved domains, we submitted the corresponding protein sequences to multiple domain structure databases, including InterPro, SMART (http://smart.embl-heidelberg.de, accessed on 18 November 2024), and Conserved Domain Database (CDD, https://www.ncbi.nlm.nih.gov/Structure/cdd/cdd.shtml, accessed on 18 November 2024).

### 4.3. Chromosome Distribution Analysis of ZmLOX Genes

To investigate the genomic distribution of *ZmLOX* genes, we retrieved data on their chromosomal locations and the lengths of all 10 maize chromosomes from the maize genome annotation file. Using TBtools version 1.098765, we performed data processing and generated visual representations of the results.

### 4.4. Motif and Domain Analysis of ZmLOX Proteins

The conserved motifs of ZmLOX proteins were identified using MEME software (v4.12) based on their protein sequences. The parameters were set as follows: a maximum motif length of 100 amino acids, a minimum motif length of 6 amino acids, 15 motifs identified, and 10,000 iterations [39]. Information on conserved domains was retrieved from the CDD database.

### 4.5. Gene Structure Analysis of ZmLOX Genes

The coding sequences (CDSs) and untranslated regions (UTRs) of maize *ZmLOX* family genes were identified based on the maize genome annotation files. These elements were analyzed and visualized using the ggplot2 (v3.5.1) and gggenomes (v1.01) packages.

### 4.6. Phylogenetic Analysis of LOX Family Members

Protein sequences of LOX family members from maize, Arabidopsis, and rice were obtained from the Phytozome database. A phylogenetic tree was constructed using the Neighbor-Joining method [40], with Clustal W employed for sequence alignment. The parameters included the Poisson substitution model and a bootstrap value of 1000. We employed the ggtree package to generate a graphical representation of LOX family evolutionary relationships [41].

### 4.7. Collinearity Analysis of Maize LOX Family Genes

Genome sequence files and gene structure annotation files for maize, Arabidopsis, and rice were retrieved from the Phytozome database. Duplication events within the *ZmLOX* family were analyzed by aligning the maize whole-genome sequence using TBtools software. Synteny relationships of *LOX* genes among maize, Arabidopsis, and rice were determined by comparing the genome sequences of Arabidopsis and rice with that of maize using TBtools and jcvi [42].

### 4.8. Cis-Acting Element Analysis of ZmLOX Gene Promoters

To identify potential regulatory elements influencing ZmLOX gene expression, we extracted the 1500 bp sequences upstream of the coding DNA sequence (CDS) for each ZmLOX gene from the maize genome database. These putative promoter regions were then submitted to the PlantCARE database (http://bioinformatics.psb.ugent.be/webtools/plantcare/html, accessed on 18 November 2024) for in silico prediction of cis-acting elements.

### 4.9. GO and KEGG Analysis

To investigate the potential functions of *ZmLOX* genes, we conducted functional annotation using the eggNOG-mapper tool [43]. This tool assigns Gene Ontology (GO) terms and Kyoto Encyclopedia of Genes and Genomes (KEGG) pathways to gene products. The resulting annotations were statistically summarized and visualized using the clusterProfiler package in R [44]. This analysis provided insights into the molecular functions, biological processes, cellular components, and metabolic pathways associated with *ZmLOX* genes.

### 4.10. PPI Network Analysis

For protein–protein interaction (PPI) network analysis, all ZmLOX protein sequences were submitted to the STRING database (https://string-db.org/, accessed on 20 November 2024) with the species set to maize (*Z. mays*). Potential interacting proteins were identified, and the results were downloaded. Proteins with the top 40 confidence scores were selected, and the PPI network was visualized using the ggraph (v2.1.0) package.

### 4.11. Expression Analysis of ZmLOX Genes

Transcriptome data (PRJNA1090631) were obtained from the NCBI database (https://www.ncbi.nlm.nih.gov/, accessed on 20 November 2024) to analyze the expression patterns of *ZmLOX* genes under cold stress conditions. Gene expression quantification was performed using the featureCounts package [45], while differential expression analysis was carried out with Trinity [46]. Volcano plots depicting differentially expressed genes were created using the ggplot2 package, and heatmaps of gene expression were visualized with the pheatmap (v1.0.12) package.

### 4.12. qRT-PCR Analysis

To analyze the expression patterns of *ZmLOX* genes, we first isolated total RNA from maize leaves using the RNA Mini Kit (Youcan Bio, Shanghai, China). Reverse transcription was performed utilizing a cDNA synthesis kit. We designed qRT-PCR primers specific to each *ZmLOX* gene using the NCBI Primer Design tool, and the primer sequences are listed in Table S1. The qRT-PCR assays were performed using qPCR SYBR Green Master Mix (Youcan Bio, Shanghai, China), with a reaction protocol consisting of an initial denaturation step at 95 °C for 5 min, followed by 40 cycles of 95 °C for 10 s, 60 °C for 20 s, and 72 °C for 20 s. We determined the relative expression levels of *ZmLOX* genes using the 2^−∆∆Ct^ method, with the maize actin gene serving as an internal reference for normalization [47]. All experiments were performed in three biological replicates for each sample.

### 4.13. Statistical Analyses

All statistical analyses in this study were performed using SPSS software (version 22). Experimental data were tested for significance using a one-way or two-way analysis of variance (ANOVA), depending on the experimental design. Post hoc comparisons were conducted using Tukey’s HSD (Honestly Significant Difference) test for a one-way ANOVA or Bonferroni correction for a two-way ANOVA, to identify significant differences between groups. A *p*-value of less than 0.05 was considered statistically significant. Data are expressed as mean ± standard deviation (SD) unless otherwise specified.

## 5. Conclusions

This study identified and characterized 13 *ZmLOX* genes in maize, uncovering their evolutionary conservation, structural features, and roles in stress responses. Promoter analyses revealed *cis*-acting elements linked to growth, light response, hormone signaling, and stress adaptation, suggesting diverse biological functions. Functional enrichment and PPI analyses highlighted the involvement of *ZmLOX* genes in jasmonic acid metabolism, lipid signaling, and photosynthesis, with ZmLOX proteins acting as regulatory hubs in stress pathways. Expression profiling and qRT-PCR confirmed stress-specific expression patterns, with *ZmLOX2* and *ZmLOX13* playing key roles in drought and cold tolerance. These findings provide a basis for leveraging *ZmLOX* genes to enhance maize resilience to abiotic stresses.

## Figures and Tables

**Table 1:** The information on *ZmLOX* gene family members.

Name	ID	Chr	Start	End	MW (KDa)	Length	Hydrophobicity	pI	Cell
ZmLOX1	Zm00001d027893	1	16948608	16955122	108.27	968	−0.33	8.59	Cytoplasm
ZmLOX2	Zm00001d031449	1	190316537	190321767	105.33	956	−0.33	6.73	Chloroplast
ZmLOX3	Zm00001d033623	1	269047817	269052874	96.73	867	−0.32	6.06	Cytoplasm
ZmLOX4	Zm00001d033624	1	269056560	269072120	100.36	887	−0.45	6.63	Cytoplasm
ZmLOX5	Zm00001d002000	2	4150293	4154404	98.66	904	−0.29	6.00	Cytoplasm
ZmLOX6	Zm00001d003533	2	47105187	47109372	105.31	941	−0.44	7.28	Cytoplasm
ZmLOX7	Zm00001d041204	3	105356377	105359732	95.99	850	−0.44	8.22	Cytoplasm
ZmLOX8	Zm00001d042540	3	171277704	171281453	100.75	892	−0.37	6.68	Cytoplasm
ZmLOX9	Zm00001d042541	3	171421717	171425401	99.33	884	−0.34	6.81	Cytoplasm
ZmLOX10	Zm00001d053675	4	238805319	238809230	102.06	905	−0.39	6.54	Chloroplast
ZmLOX11	Zm00001d013493	5	12701180	12706156	100.36	887	−0.43	6.83	Cytoplasm
ZmLOX12	Zm00001d015852	5	126372618	126376084	102.87	911	−0.39	7.04	Chloroplast
ZmLOX13	Zm00001d025524	10	121268606	121272616	103.6	929	−0.42	7.14	Cytoplasm

## Data Availability

The raw sequencing reads were deposited at the National Center for Biotechnology Information Sequence Read Archive (https://submit.ncbi.nlm.nih.gov/subs/sra/, accessed on 15 September 2024) under Project Accession Number PRJNA1090631.

## Supplementary Materials

The following supporting information can be downloaded at https://www.mdpi.com/article/10.3390/genes16010099/s1. Table S1. qRT-PCR primers for *ZmLOXs.*
